# The Impact of Menthol Cigarettes on Smoking Initiation among Non-Smoking Young Females in Japan

**DOI:** 10.3390/ijerph8010001

**Published:** 2010-12-23

**Authors:** Gregory N. Connolly, Ilan Behm, Yoneatsu Osaki, Geoffrey F. Wayne

**Affiliations:** 1 Center for Global Tobacco Control, Harvard School of Public Health, 677 Huntington Ave, Landmark Center, 3rd Floor East, Boston, MA 02215, USA; E-Mail: ferriswayne@gmail.com (G.F.W.); 2 Department of Environmental and Preventative Medicine, Faculty of Medicine, Tottori University, Nishimachi 86, Yonago, Tottori 683-8503, Japan; E-Mail: yoneatsu@med.tottori-u.ac.jp (Y.O.)

**Keywords:** female-smoking, smokers, initiation, menthol, Japan

## Abstract

Japan presents an excellent case-study of a nation with low female smoking rates and a negligible menthol market which changed after the cigarette market was opened to foreign competition. Internal tobacco industry documents demonstrate the intent of tobacco manufacturers to increase initiation among young females through development and marketing of menthol brands. Japanese menthol market share rose rapidly from less than 1% in 1980 to 20% in 2008. Menthol brand use was dominated by younger and female smokers, in contrast with non-menthol brands which were used primarily by male smokers. Nationally representative surveys confirm industry surveys of brand use and provide further evidence of the end results of the tobacco industry’s actions—increased female smoking in Japan. These findings suggest that female populations may be encouraged to initiate into smoking, particularly in developing nations or where female smoking rates remain low, if the tobacco industry can successfully tailor brands to them. The Japanese experience provides a warning to public health officials who wish to prevent smoking initiation among young females.

## 1. Introduction

The smoking rate among Japanese adults peaked in the 1960s (47.1% in 1965) and decreased to 34.6% by 1997. The decline, however, mainly reflects a decrease in male smoking rates from 83.2% in 1965 to 57.5% in 1997, while smoking rates among women remained constant (15.7% in 1965 and 14.5% in 1997) [1]. More recent data indicates that while smoking rates continue to decline among adult men (45.3% in 2009), rates among adult women are rising (to 16.1% in 2004, and 18.2% in 2009) [2]. A survey of adolescent smokers conducted in 1996 and 2000 indicates a similar pattern of rising use among high school aged girls. As reported by Osaki *et al.*, although prevalence among Japanese girls remained much lower than that among boys (8.2% versus 25.9% daily smokers in grade 12), prevalence among girls increased in 2000 [3].

**Figure 1:**
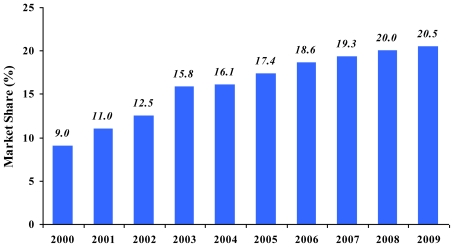
Menthol share of the Japanese cigarette market (2000–2009); data from [2,5].

Menthol brands have historically played an insignificant role within the Japanese market, with less than 1% of the overall market as of 1980 [4]. However, current estimates demonstrate a sharply rising trend in adult menthol use in Japan (Figure 1). Among adolescent smokers (both boys and girls), survey data indicates rapid growth in menthol brand use in Japan between 1996 and 2000, coincident with the rise in overall use [3].

The introduction and marketing of women-targeted brands capitalizes on social changes and promotes further initiation of women into smoking. Carpenter *et al.* [6] describes industry targeting of women in the United States with cigarette brands that appeal directly to female preferences including style (Slims), safety (Lights), and ease of inhalation (Menthol). Carpenter also describes how female brands can serve as entry products and that once women have accustomed to smoking, many gradually transition to more gender-neutral brands.

Among the design characteristics that have appealed to women, menthol has important chemosensory properties including masking of irritation, which facilitates inhalation of tobacco smoke [7]. Menthol activates “cold” receptors called TRM8 receptors located in the olfactory and trigeminal nerves producing perceptions of coolness in the upper respiratory tract. This sense of “cooling” makes the inhalation of irritants such as nicotine or other smoke constituents perceived less irritating and less harmful [8,9]. Concerns about menthol cigarette use among younger smokers in the United States have been raised following evidence that menthol may ease initiation and enable beginners to progress to long term use [10–14].

In many countries with low female smoking, such as Russia and Poland, in which the role of women in society and social stigmas associated with female smoking are evolving, the tobacco industry is actively promoting new menthol brands [15,16]. If successful, the introduction and promotion of menthol brands may be a key to smoking initiation for some of the billions of women worldwide who do not smoke.

Japan presents an excellent case-study of a nation with low female smoking rates and a negligible menthol market which changed after the cigarette market was opened to foreign competition. Until the late 1980s, Japan was dominated by Japan Tobacco International (JTI), a government monopoly. US trade threats forced foreign entry into the cigarette market in 1986 and foreign manufacturers make up more than a quarter of the market today [2].

The changes in the Japanese market provide an opportunity to study how the introduction and promotion of menthol brands contributed to female smoking initiation in Japan. The current study examines the available evidence regarding the development and growth of the menthol market in Japan, including tobacco industry intent, introduction and marketing of menthol brands, and the impact on initiation and use among young female smokers from published survey data.

## 2. Experimental Section

The research question was explored via analysis and synthesis of three primary data sources: 1) internal tobacco industry documents, 2) adolescent and 3) adult surveillance data. The internal documents provide insight into industry intent and practices, as well as historical monitoring of brand preference and use. However this data is limited in terms of reliability and findings are effectively unavailable after the year 2000. Adolescent surveillance data is available for 1996 and 2000, and provides confirmation of the findings from the internal documents, while supporting further quantitative analysis of the effects of menthol introduction specifically among high school aged girls. Adult (aged 20+) gender-specific surveillance data is employed to compare trends in use among adolescent girls to young and older women and to identify ongoing trends as these cohorts age.

Tobacco industry documents on intent to market menthol brands to females, marketing practices, and impact on female initiation, were drawn from the more than 10 million internal documents available as a result of tobacco litigation at the Legacy Tobacco Documents Library (http://www.legacy.library.ucsf.edu). Document searches were conducted with keyword searches combining broad terms such as “menthol”, “Japan”, “YAS” (young adult smoker), and “female”, and then increasing specificity of searches by means of brand names, projects, and other industry terms identified in initial searches. A total of 660 documents were identified as relevant to the themes of menthol brand development and use in Japan. Criteria for relevance consisted of 1) identification of strategic priorities in Japan, 2) measures of physical brand characteristics and descriptors of brand packages and marketing, 3) consumer market research including population characteristics and brand consumption. Included in these documents were large-scale, internal surveys of smoker preference and brand use which provide historical surveillance of the changing market. Interpretation of documents was conducted by two or more readers and documents were indexed and organized within a historical and thematic narrative.

Analysis was performed on a cross sectional survey of Japanese adolescents conducted in two waves, 1996 and 2000, first reported in Osaki *et al.* [3]. These surveys used a single-stage cluster sampling methodology with the unit of cluster being the sampling of schools. The survey targeted junior and senior high school students. Although menthol was not a primary focus of the original survey, the study authors noted observationally a rise in use among both boys and girls of brands with the term “menthol” in the brand name. For the current study, brand name has been cross referenced against package color and descriptors (recorded in the original survey) and a historical record of the brands available in the Japanese market in 1996 and 2000, in order to produce a more accurate categorization of menthol versus non-menthol brands. Estimated market share of individual cigarette brands was calculated by dividing the sum of cigarettes smoked for each brand by the sum for all brands. Comparisons were made across time periods (1996 and 2000) and high school versus overall market. Data were analyzed with a 2-sample test for equality of proportions using SPSS 10.0.

Further analysis was conducted using the Japanese National Health and Nutrition Survey, a nationally representative annual survey of randomly selected Japanese adults aged 20–69. The survey has been conducted annually since 1952. Female smoking prevalence was the key measure of interest and was calculated for all females by 10–year cohorts (20–29, 30–39, 40–49, 50–59 and 60–69) for years 1989–2004. Age-cohort specific trends were smoothed with three-year moving averages, to account for annual variations in survey results. Differences in changes in age-cohort specific temporal trends was assessed by annual percent changes (APCs) to ascertain the point of initial increase in smoking prevalence, using National Cancer Institute’s Joinpoint 3.4.3 software. The estimated annual percent change (APC) was tested for a statistically significant trend (with a two-sided p-value ≤0.05).

## 3. Results and Discussion

### 3.1. Internal Tobacco Industry Documents

#### 3.1.1. Intent to Target Women with Menthol Brands

Tobacco manufacturers identified women as critical to market growth in Japan [17,18], despite negative social attitudes in Japan toward female smoking [19–21].

Product wants identified among female smokers in Japan reflected the need to overcome negative social and personal attitudes of smoking including low tar delivery, less tobacco taste, and reductions in odors particularly in hair and clothing [20,22]. Brands were targeted to women by means of “feminine” attributes including slim, light, mild, box, and menthol [17].

Surveys identified menthol as a major reason for female experimentation and use [23]. Menthol cigarettes tended to be viewed as “a special type” of ‘light’ cigarette [20] and were widely considered to be an exclusively female domain [17,24]. Levels of menthol were typically much lower in Japanese cigarettes relative to US cigarettes [25]. Reasons for menthol use included a perceived lack of the typical tar-like smell of cigarettes [20].

Manufacturers recognized the appeal of menthol not only among women but increasingly among first time, young smokers. A 1990 PMI review of menthol brands observed that the low tar menthol brand Salem was generally successful in:

…attracting a higher proportion of young adult women, in markets where the incidence of young adult female smokers is growing as women become more emancipated. In Japan there are strong indications that the Salem franchise is attracting the same kind of smokers [26].

This reflected a larger trend throughout Asia, including Hong Kong and Malaysia, where foreign menthol brands led by RJR’s Salem “became the natural selection for YAUS (young adult urban smoker) smokers because of its lighter menthol taste compared with full strength [non-menthol] brands” [27]. (see Table 1)

#### 3.1.2. Introduction and Marketing of Menthol Brands

Foreign tobacco companies marketed extensively in Japan during the period immediately following the removal of trade restrictions in order to expand brand awareness and share. Of the 60 billion yen spent on tobacco advertising in Japan in 1993, half was by foreign manufacturers [36]. Marketing included television ads, event sponsorships, and celebrity endorsements, as previously documented in Lambert *et al.* [37]. The foreign brand segment expanded rapidly, growing from 3.9% in 1986, to 21.2% in 1995 [38].

Marketing expenditures for menthol brands made up only a small fraction of total spending [29,36] and companies appear to have relied heavily on promoting the brand family and not the menthol sub-brand [17,27,39], possibly allowing the properties of the menthol product (taste, coolness) to serve as the main marketing tool. Despite reduced spending, growth within the menthol segment consistently exceeded growth of the total market and was driven by foreign brands including Salem, Virginia Slims, Kool, and Marlboro Menthol [40]. Foreign brands made up 16% of all menthol cigarettes sold in 1981 (or less than 0.1% of the total market), but 58% of the menthol segment in 1992 [4,36]. Menthol brands had grown to 5% of the total market by 1995, 10% by 2000, and 20% by 2008 [15,36,40].

Menthol brands continued to be targeted primarily toward female smokers, with Kool, a high menthol, tar and nicotine cigarette, being the major exception targeted towards male current smokers [20,41]. Brand extensions were a key strategy for introducing new menthol products, for example, Philip Morris introduced Virginia Slims Ultra Light Menthol, capitalizing on the preference of female smokers for lower tar brands with low nicotine impact combined with “coolness” derived from menthol [18,42,43]. Salem Pianissimo (a menthol sub-brand of Salem) was introduced in 1995 as a “low smoke and low odor” brand targeted at women. By the end of 1996 it was the third largest brand in the menthol segment [44].

**Figure 2:**
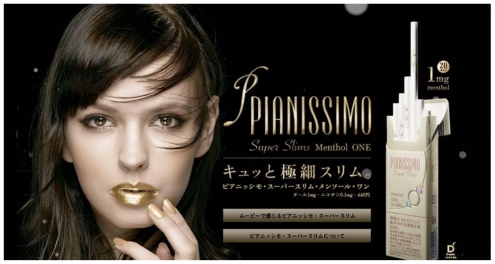
Japanese advertisement for menthol cigarette ‘*Salem Pianissimo Super Slims Menthol One’.* (photo courtesy of Dr. Yumiko Mochizuki-Kobayashi—National Cancer Center Research Institute Japan—2010)

Salem and Virginia Slims dominated menthol sales in Japan through the early 1990s. In 1992, Salem Lights made up 23% of all menthol sold in Japan, while Virginia Slim Lights Menthol (VSLM) had a 20% share [45]. The only major domestic menthol brand, Sometime Lights, held a comparable share of the menthol market (~22%) in 1992, but declined to 12% by 1995 [45]. Menthol extensions of other domestic brands (e.g., Mild Seven, Frontier) were minor players in the menthol market as was the foreign brand Kool, with each holding less than 5% of all menthol sales [45]. The total Salem family had grown to 2.4% of the total Japanese market in 1995, and 4.2% projected for 1996 [46]. However, the profile of the Salem brand family shifted with the introduction of extensions such as Pianissimo and Salem Slim Lights (see Figure 2). In 1992, fifty-five percent of Salem smokers were in their 20’s. By 1995 only 39% were in their 20’s and 43% were aged 30–39 [46].

Marlboro Lights Menthol were launched in 1993, and Marlboro Menthol in 1995. Both brands were initially positioned against Kool as a “men’s menthol” and quickly gained market share [36]. Marlboro menthol sales soon exceeded its non-menthol sales [47,48], despite the fact that marketing for menthol was less than 2% of overall brand spending [36].

By 1998 Marlboro Lights Menthol had taken the leading position within the menthol segment and Marlboro Menthol brands were “dramatically gaining the share [sic] among young females” [49]. By 2000, Marlboro held 35% of the menthol market in Japan, and Marlboro Lights Menthol held more than a quarter of the menthol market, double the share of any other single menthol brand [50,51].

A PM analysis into these brand dynamics separated the menthol market into “feminine” and “not-feminine”, and noted that Marlboro had grown successful among younger Japanese women precisely because it was not considered exclusively feminine and yet was perceived as acceptable among women (see Table 2) [49]. Among the significant brand differences, Marlboro Menthol Lights were King size while major Salem and VSLM entries were longer length and slimmer cigarettes. Marlboro also had higher per puff “tar” and nicotine, and a relatively low level of menthol (see Table 3) [52]. Decreasing femininity-image coincided with increasing per puff nicotine (strength) and decreasing menthol (ease) in the rod. Thus, the success of Marlboro Menthol represented an important shift for young women further into the smoking mainstream.

#### 3.1.3. Impact of Menthol Brands on Trial and Use

Menthol brands proved uniquely successful in reaching female smokers in Japan. In a 1989 Philip Morris survey (N = 50,000), Virginia Slim Lights Menthol (VSLM) and Salem Lights profiled as the most female-oriented brands on the Japanese market, with 76% of VSLM smokers and 38% of Salem Lights smokers identified as women [23].

An ongoing wave-based survey of smokers (N = 4,000) conducted for Brown & Williamson in 1990 found that menthol accounted for 2% of regular brands smoked by men, and 10% of regular brands smoked by women in 1990 [53]. In 1996 the survey (N = 3,000) showed that male smokers accounted for 81.5% of the non-menthol market, and female smokers 18.5%, matching their respective share of the total market. Yet women comprised 61% of the menthol market to men’s 39%. Half of these female menthol smokers were in the 20–29 year old age group, and 45% of all menthol smokers (male and female) were aged 20–29, compared with 22.5% of all non-menthol smokers [36].

Foreign menthol brands were more successful than domestic menthol brands in attracting younger smokers, and profiled younger than non-menthol import brands. In a 1985 Philip Morris study on smoking profiles for major import and domestic brands, smokers aged 20–29 made up 24% or the cigarette market, but 62% of Salem and 49% of VSLM, the two most popular import menthol brands [54]. A 1994 PMI continuous tracking survey (N = 30,000) identified VSLM with 14% of the 20–24 year old female market, and Salem Lights with 7% (ranked second and fourth behind Mild Seven Lights and Mild Seven) versus total share among women of 6% and 2%, respectively [55].

The 1989 Philip Morris survey identified the average age for smokers of individual brand styles [23]. VSLM (aged 28.5) and Salem Lights (27.7) were two of only three brands that profiled as under age 30, with nearly three-quarters (71.6% and 72.4%, respectively) of each brands’ smokers aged 20–29. Major domestic brands Mild Seven (40.3) and Caster (38%) were among those used by the oldest population (both non-menthol).

A 1992 Philip Morris intercept survey of Japanese smokers (N = 20,996) identified the three youngest Japanese brands as VSLM (29.3), Marlboro Lights (29.9), and Salem Light (30.7) [56]. The survey also identified the percent of smokers of each brand that were first time smokers, and the brand with the highest rate of first time smokers was VSLM (11.3%, N = 345). Philip Morris also conducted ongoing surveys on brand use among the YAS (young adult smoker) segment of smokers aged 18–24. By 1995, Marlboro had grown to become the second leading brand among YAS (12.7%), behind only Mild Seven (34.5%) [57]. Marlboro (combining both menthol and non-menthol sub-brands) became the leading brand among YAS in 2000 [50].

### 3.2. Independent Survey Data

#### 3.2.1. Adolescent Smoking Survey

**Figure 3:**
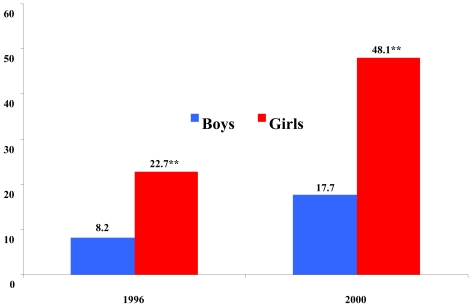
Share of menthol brand preference among Japanese adolescents by gender (1996 & 2000) (data from [3]). **Two sample test for equality of proportions significant difference among gender groups at the 95% significance level (p < .001 in 1996 & p < .001 in 2000).

Surveillance data collected among a sample of high school students in Japan indicates a significantly higher rate of menthol use among high school aged girls versus boys in both 1996 (p < 0.01) and 2000 (p < 0.01) (see Figure 3). By 2000, approximately half of all female adolescent smokers were smokers of menthol brands. Menthol use among adolescents (both male and female) was significantly higher than use in the overall national population in both time periods. Published results of this data set indicate that current smoking prevalence among females in their last year of junior-high school (grade 9) increased from 5.5% in 1996 to 6.9% in 2000 [3].

Among high school girls, top brands in 1996 included Marlboro Lights Menthol (ranked 5th), Marlboro Menthol (8th), Virginia Slims Menthol (9th), Virginia Slims Light Menthol (14th), Kool (15th), and Salem (17th). By 2000, Marlboro Lights Menthol and Marlboro Menthol ranked 1st and 2nd in brand use among girls.

#### 3.2.2. Japanese National Health and Nutrition Survey

**Figure 4:**
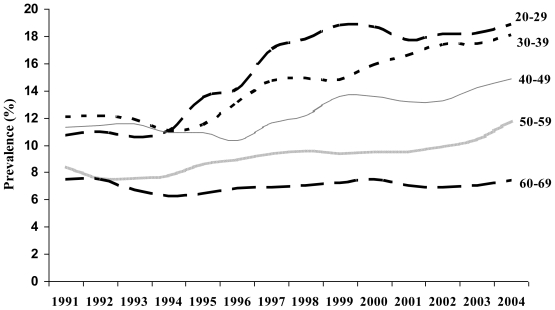
Japanese female smoking prevalence—Three year moving average of female Japanese smoking prevalence using 10-year age groups.

The Japanese National Health and Nutrition Survey provides data on adult smoking prevalence (age 20+). Although this data does not indicate specific brand preference or menthol use, these data describe a rise in smoking prevalence among Japanese women driven primarily by new young smokers. Among Japanese females ages 20–69, smoking prevalence significantly increased (slope = .589, p < .05) over the period 1989 to 2004. Among the youngest cohort, aged 20–29, smoking prevalence increased by a higher magnitude (slope = .713, p < .05). Joinpoints in temporal trends were evident by age-group. An initial increase was seen in smoking prevalence among the 20–29 year-old female cohort in 1993 with an APC of 12.01 from 1993 through 1998. Smoking prevalence among the 30–39 year old cohort began to increase in 1994 with an APC of 9.26 from 1994–1997 and the 40–49 year old female cohort had increasing smoking prevalence beginning in 1996 with an APC of 3.86 from 1996–2004 (see Figure 4).

### 3.3. Discussion

Japan provides an excellent case-study on the impact of the introduction and marketing of menthol brands to young women and initiation into smoking. Smoking prevalence in Japan has been declining among men for decades, but rates of smoking among women have recently increased. The internal tobacco documents demonstrate the intent of tobacco manufacturers to increase initiation among young females through development and marketing of menthol brands. Adolescent survey data provides evidence that the uptake of smoking among high school aged girls in Japan is largely via menthol brands [3]. Adult survey data, though not segmented by brand type (e.g., menthol), indicates that the increased uptake of smoking among young girls has translated to rising smoking rates among young women as these cohorts age.

Previous studies have linked menthol brands to initiation, and trial as well as regular use [11,12]. The current findings suggest that female populations may be encouraged to initiate into smoking, particularly in developing nations or where female smoking rates remain low, if the tobacco industry can successfully tailor brands to them.

Direct menthol advertising in Japan was minimal and companies relied on advertising brand families and menthol characteristics and packaging to serve as a marketing vehicle. Menthol use also increased among young male smokers, although that was not the focus of this study and warrants further research. Young female smokers progressed from lighter, exclusively “feminine” menthol products to menthol products (e.g., Marlboro Menthol) with higher “tar” and nicotine yield that were acceptable to both male and female smokers. This dynamic has been previously reported with respect to trends among women smokers in the US [6].

Following their success within the adolescent market, menthol brands showed accelerated gains within the broader market, again repeating market trends demonstrated elsewhere [7]. Menthol cigarette use in Japan has continued to rise linearly achieving a 20% share in 2008 [2].

The growth of menthol in Japan parallels the introduction and growth of the menthol brand segment in the US Although menthol brands were first introduced there in the 1930s, menthol accounted for less than 1% of the US market prior to the 1950s, following which the segment expanded rapidly to a quarter of the market by the 1970’s. The growth was driven largely by women and youth. A 1991 review by Philip Morris summarizes the reason for menthol’s success: “The market was saturated with new smokers whose tastes were not well established and smokers—women—who, over the preceding and following years, embraced any concept which represented a further step away from what tobacco use had traditionally been.” [30]. Indeed, the success of menthol among both youth and women in the US continues to the present [11,12].

The Japan experience suggests the importance of brand targeting as a vehicle to capitalize on social changes and encourage initiation and use among a historically non-smoking population. Product design characteristics such as menthol which exhibit significant chemosensory effects increase the appeal of the product and ease the path toward long-term smoking. This is a lesson the tobacco industry has learned well—according to Philip Morris (1990):

“… Japan is a prime example of us taking a proactive situation or a proactive involvement in the market place…..The decision that was taken early when that market opened up was to get in there and develop products designed for the Japanese market place…getting products into that market, and going through evaluation procedures to understand the sensory characteristics of the Japanese consumer, allowed us to understand that market place better. And again, that bottom line, that base, was understanding the sensory characteristics….” [58]

### 3.4. Study Strengths & Limitations

The synthesis of various data types and sources serves to cross-validate the finding that the marketing of menthol brands in Japan contributed to an increase in female smoking prevalence. However, the lack of menthol-specific prevalence data is a limitation of this study. Future surveillance surveys should obtain menthol-specific prevalence rates. Additionally, the increase in smoking among Japanese females took place during a time in which women were entering the workforce and increasing disposable income, as cultural barriers to female smoking were declining, and as competition in the market led to reduced tariffs and expanded advertising. All of these factors could have contributed to an increase in female smoking. However, women did not merely take up brands popular with the general population. They generally began with brands tailored to them by the tobacco industry.

## 4. Conclusions

The experience in Japan illustrates how different menthol brands were continually introduced and intentionally marketed to young non-smoking females in Japan when the market was opened to competition. Chemosensory properties of menthol ease initiation and may play an important role in their attractiveness among females. The combination of social changes towards women’s smoking and the introduction of menthol brands appears to have contributed to smoking among non-smoking females in Japan. Given the very low prevalence of female smoking in many developing nations with developing markets and changing social norms, multinational tobacco companies may seek to repeat their success in Japan in other nations, such as Russia. The Japanese experience provides a warning to public health officials who wish to prevent smoking initiation among young females and smoking generally.

## Figures and Tables

**Table 1:** Quotes from internal tobacco industry documents describing the potential appeal of menthol brands and targeting of young women (data from [20,28–35]).

Theme	Source	Quotes

Perception of menthol brands	BW, 1994	“The menthol segment in Japan has long been identified with ‘lightness,’ being dominated by Virginia Slim Lights Menthol, Salem Lights, Salem Slim Lights, and Sometime Lights.” [28]
	PM, 1991	“The desire for a “lighter” cigarette was an important underlying motive for switching to menthol cigarettes.” [20]
	PM, 1991	[From focus group testing ]: “More like candy than ordinary cigarettes/Ordinary cigarettes and menthol cigarettes are completely different.” [20]
	PM, 1991	“Female menthol smokers were generally more satisfied with menthol cigarettes than male menthol smokers, as current menthol cigarettes fulfilled a greater range of needs for females (particularly psychological needs, such as fashionableness, expression of femininity).” [20]

Menthol attracts newer smokers	RJR, 1996	“Movement to low tar and menthol is partially “trendy/modern” and partially personal concern. Also females/young adults coming to market now often start with low tar or menthol brands.” [29]
	PM, 1991	“I strongly suspect that, as was true in the US, especially for Kool and partially for Salem, where the menthol share is growing, it is not doing so by attracting smokers with established tastes, but by attracting new smokers…. new smokers may be young beginning smokers, newly “liberated” females of any age, or people of any age just beginning to acquire the economic status required to smoke manufactured cigarettes.” [30]
	PM, 1988	“…these younger smokers also show considerable interest in menthol cigarettes”. [31]
	PM, 1985	“The menthol market could develop quite rapidly as there is considerable interest in cigarettes with a cool refreshing flavor. Its main source of business is likely to be new younger smokers of both sexes.”[32]

Intent to grow the menthol segment and reach young women	BAT, 1996	“Given the growing importance of females and the long-term growth history of menthol slims, it seems advisable to try to participate in this slims segment.” [33]
	PM, 1995	“The growing young adult female segment in Japan has been identified as a market opportunity for further growth.” [34]
	PM, 1993	“Menthol products, though still small in volume terms, represent one of the strongest growth categories in Asia, particularly outside traditionally menthol markets like the Philippines.” [35]
	PM, 1991	“Menthol should have potential in any market in which the percentage of female smokers is growing.” [30]

**Table 2:** PMI analysis of the image profile of major menthol brand styles in Japan, 1998 (adapted from [49]).

	Salem Pianissimo (100’s slim)	VSLM (100’s slim)	Marlboro Lts. M (KS)
	“Major” and “Widely” Acceptable		
*Basic Value*	“low smoke/low odor” and “1mg tar” cigarette for women	“basic” women’s cigarette	“KS” and “higher tar” cigarette which even women can smoke
*Image value*	Feminine; Natural/Soft women	Feminine; Independent women	Not feminine; Women with strong personality

**Table 3:** Reverse engineering cigarette parameters of japanese cigarettes—1999 British American tobacco (data from [52]).

Parameter	Salem Pianissimo (100’s slim)	VSLM (100’s slim)	Marlboro Lights Menthol (KS)
*Nicotine/Puff (mg) (ISO)*	0.01	0.05	0.08
*Tar/Puff (mg) (ISO)*	0.15	0.63	1.13
*Menthol (Rod %)*	1.28	0.82	0.57
